# Enhanced Oral Pre-exposure Prophylaxis (PrEP) Implementation for Ugandan Fisherfolk: Pilot Intervention Outcomes

**DOI:** 10.1007/s10461-024-04432-w

**Published:** 2024-07-19

**Authors:** Laura M. Bogart, William Musoke, Christopher Semei Mukama, Stella Allupo, David J. Klein, Abdulrazake Sejjemba, Simon Mwima, Herbert Kadama, Ronald Mulebeke, Rakesh Pandey, Zachary Wagner, Barbara Mukasa, Rhoda K. Wanyenze

**Affiliations:** 1 RAND Corporation, 1776 Main Street, P.O. Box 2138, Santa Monica, CA 90407-2138 USA; 2 Department of Psychiatry, Charles R. Drew University of Medicine and Science, Los Angeles, CA USA; 3 Mildmay Uganda, Kampala, Uganda; 4 School of Social Work, University of Illinois at Urbana Champagne, Urbana, IL USA; 5 Ministry of Health, Republic of Uganda, Kampala, Uganda; 6 Makerere University School of Public Health, Kampala, Uganda

**Keywords:** Fisherfolk, HIV, Implementation science, Pre-exposure prophylaxis (PrEP), Uganda

## Abstract

Mobile populations such as fisherfolk show high HIV incidence and prevalence. We pilot-tested implementation strategies to enhance pre-exposure prophylaxis (PrEP) uptake and adherence in the context of healthcare outreach events in two mainland fisherfolk communities on Lake Victoria, Uganda from September 2021 to February 2022. The implementation strategies included PrEP adherence supporters (selected from PrEP users’ social networks), community workshops (to address misconceptions and stigma, and empower PrEP advocacy), and check-in calls (including refill reminders). PrEP medical records data were collected from 6-months pre-intervention to 6-months post-intervention. Qualitative interviews with 20 PrEP users (10 who continued, 10 who discontinued), 9 adherence supporters, and 7 key partners (providers, community leaders) explored acceptability. Percentages of PrEP initiators (of those eligible) were significantly higher during the intervention (96.5%) than 6-months before the intervention (84.5%), p < 0.0001; percentages of PrEP users who persisted (i.e., possessed a refill) 6-months post-initiation (47.9% vs. 6.7%) and had at least 80% PrEP coverage (based on their medication possession ratio) from the initiation date to 6-months later (35.9% vs. 0%) were higher during versus pre-intervention, p < 0.0001. A comparison fisherfolk community with better healthcare access had lower uptake (78.3%; p < 0.0001) and persistence at 6-months (34.0%; p < 0.001), but higher coverage during the intervention period (70.4%; p < 0.0001). Qualitative data suggested the strategies promoted PrEP use through reduced stigma and misconceptions. The intervention bundle cost was $223.95, $172.98, and $94.66 for each additional person for PrEP initiation, persistence, and coverage, respectively. Enhanced community-based PrEP implementation that fosters a supportive community environment can improve PrEP use in mobile populations without easy access to healthcare. (NCT05084716).

## Introduction

In Uganda, HIV incidence and prevalence among fisherfolk are estimated to be at least 3–4 times higher than the general population incidence of 0.29% and prevalence of 5.8% (in ages 15 and older) [1–4], in part due to their lower access to HIV prevention and treatment services [5]. About 600,000 Ugandans were estimated to be taking oral pre-exposure prophylaxis (PrEP) as of July, 2024, which is a small fraction of the population aged 15 and older who may be eligible for PrEP (estimated to be ~ 27,000,000) [6]. Although Uganda has made progress toward the 95–95–95 UNAIDS goals, further prevention efforts are needed given 2020–2021 Uganda Population-based HIV Impact Assessment statistics of 90% knowing their status, 84% on antiretroviral therapy (ART), and 79% virally suppressed [4].

Fisherfolk are “at substantial risk” for HIV infection and thus eligible for PrEP under the Uganda Ministry of Health’s 2022 PrEP technical guidelines [7]. However, consistent with updated conceptualizations of the Consolidated Framework for Implementation Research (CFIR) [8, 9], and similar to determinants of PrEP use for other populations in sub-Saharan Africa [10–12], many Ugandan fisherfolk lack access to PrEP because of contextual barriers at multiple levels related to the external environment (outer setting), healthcare system (inner setting), individual fisherfolk (PrEP user), and PrEP (innovation) levels. In the external environment, high levels of stigma around PrEP and HIV, gender-based violence, and alcohol use in fisherfolk communities contribute to low PrEP use [3, 13–16]. At the healthcare system level, healthcare facilities are commonly far from where East African fisherfolk live, work, and travel [13, 17, 18], and providers may have insufficient staffing and travel support to provide PrEP in remote communities [19]. Barriers among fisherfolk include mobility (to follow fish migrations and move between urban and rural areas, and from islands to the mainland, to sell fish), competing needs for work and child care, PrEP misconceptions, low perceived risk, and poverty-related issues such as food insecurity and lack of money for transport to get refills [19–23]. At the PrEP level, fisherfolk perceive high pill burden, as taking a daily pill may be challenging with irregular work schedules and lack of private places to store pills during work (e.g., in boats over weeks or months); they also may fear side effects and anticipate partner conflict and stigma because PrEP medications have similar packaging as HIV treatment [19]. As a result of these barriers, fisherfolk underutilize antiretroviral medications for PrEP as well as HIV treatment [5, 24], increasing transmission risk [25].

Fisherfolk show high acceptability of (i.e., preference for) PrEP over other HIV prevention methods [26, 27] but low continuation over time (also called PrEP “persistence”), similar to other populations in sub-Saharan Africa [21, 28]. However, most existing PrEP delivery models are tailored for key populations (e.g., sex workers, adolescent girls and young women, sexual and gender minority individuals) rather than whole-communities with high HIV prevalence and incidence that contain key population groups as well as general population members [23, 29]. Thus, in the present study, we conducted a mixed-methods pilot-test of the acceptability and preliminary effectiveness and cost-effectiveness of community-based PrEP implementation strategies for Ugandan fisherfolk, including healthcare outreach events, PrEP adherence supporters, community workshops, and check-in calls (described in Table 1). We hypothesized that this combination of implementation strategies would lead to higher PrEP uptake (of those eligible for PrEP) and higher PrEP persistence over 6 months.

**Table 1:** PrEP implementation strategies for Fisherfolk: description, ERIC (expert recommendations for implementing change) strategy, and CFIR (consolidated framework for implementation research) barrier addressed

*Healthcare outreach event*: Mobile clinic program to deliver HIV testing and treatment, PrEP, and basic healthcare needs in temporary/community venues	
ERIC strategy ∙ Conduct educational meetings ∙ Increase demand ∙ Intervene with consumers to enhance uptake and adherence ∙ Inform local opinion leaders ∙ Provide local technical assistance	CFIR-related barrier addressed ∙ External Setting (stigma): community sensitization (with local providers/leaders); Primary health and HIV care provided together ∙ Healthcare System (far away facilities) and Individual Patient (mobility): outreach events scheduled at convenient times and occur in communities where fisherfolk live and work
*Adherence supporters*: Healthcare workers trained to guide PrEP users to select adherence supporter, to remind them about daily adherence and refills	
ERIC strategy ∙ Involve consumers and family members ∙ Intervene with consumers to enhance uptake and adherence	CFIR-related barrier addressed ∙ External Setting (stigma): leverages power of social support in improving adherence and reducing stigma ∙ Individual Provider (low knowledge): providers trained to provide guidance on adherence supporter selection ∙ Individual Patient (nondisclosure): increases social support, which increases motivation
*Workshops*: during events, local facility providers trained to lead community workshops to educate people thinking about, initiating, and using PrEP	
ERIC strategy ∙ Conduct educational meetings ∙ Develop educational materials ∙ Identify and prepare champions ∙ Increase demand ∙ Intervene with consumers to enhance uptake and adherence ∙ Involve consumers and family members ∙ Prepare consumers to be active participants	CFIR-related barrier addressed ∙ External Setting (stigma): open dialogue about PrEP and more people on PrEP leads to stronger norms for PrEP and more contact with PrEP users—promoting positive norms for PrEP acceptance ∙ Individual Provider (low knowledge): train local providers ∙ Individual Patient (low knowledge): educate about PrEP
*Check-in calls*: healthcare workers trained to call PrEP users 2 weeks post-initiation and 1 week before refill to answer questions, problem solve, and remind about next refill	
ERIC strategy ∙ Intervene with consumers to enhance uptake and adherence	CFIR-related barrier addressed ∙ External setting (stigma): provider support can buffer stigma and thus increase motivation ∙ Provider (low knowledge): train local providers ∙ Individual Patient (low knowledge): educate about side effects

## Methods

### Setting

This study was conducted at two mainland fisherfolk landing sites near Entebbe, Wakiso District, Lake Victoria, Uganda. The team was restricted to communities in which the medical implementing partner had service provision grants to partner with local providers, who collected PrEP-related data for their facility. The intervention community was estimated to have 13,500 residents (based on 2014 census data [30]), of whom about 7,399 were eligible for PrEP (aged 15 and older). A comparison community was selected from the same area that was approximately the same size, with a similar number of residents (14,065, with an estimated 7,709 eligible for PrEP), and with low probability of contamination and overlap with intervention community residents. However, the communities were mismatched, with critical differences that affected PrEP provision and assessment. In the comparison community, PrEP refill data were not routinely recorded pre-intervention (i.e., at baseline) in hard-copy registers at the sub-county healthcare facility, and no PrEP data were digitized. PrEP data were only available in the comparison community during the intervention period, after the team built healthcare workers’ capacity to collect and enter PrEP data. Moreover, in the comparison community, PrEP was provided through a Health Care Center III (a general outpatient clinic facility covering a sub-county) that had a less mobile client population, making refill access easier than in the intervention community, which did not have a healthcare facility.

### PrEP Implementation Strategies and Procedures

During the approximately 6-month intervention (September 2021–February 2022), in the context of healthcare outreach events, three strategies were used to enhance PrEP implementation in the intervention community: adherence supporters, community workshops, and check-in calls. These strategies, which arose from our formative research and discussions with the Uganda Ministry of Health PrEP Technical Working Group [19], were hypothesized to reduce contextual barriers to PrEP use as described by the expanded CFIR [8, 9] and are consistent with implementation strategies listed under the Expert Recommendations for Implementing Change (ERIC) project, a taxonomy of different types of methods that can be used to enhance implementation of a clinical intervention such as PrEP (see Table 1) [31].

#### Healthcare Outreach Events

During the study, at healthcare outreach events in both the intervention and comparison communities, local healthcare providers and community leaders sensitized and mobilized fisherfolk around HIV prevention and PrEP, and members of key and priority populations, including fisherfolk, were able to access HIV testing, ART for HIV treatment, and PrEP for HIV prevention at no cost through a government-funded PEPFAR program. This strategy, a well-established method for delivering HIV testing [32], was based on research in sub-Saharan Africa (including Uganda) indicating the feasibility of PrEP evaluation and initiation through community events [29, 33, 34]. Moreover, in prior research, key partners (e.g., policymakers, faith- and community-based organizations, health facilities) have suggested providing PrEP to fisherfolk through non-facility, community-based outreach events with flexible hours that include community-wide education to reduce stigma, misconceptions, and mobility barriers [35]—and in a card-sorting and ranking study in Lesotho, PrEP policymakers, healthcare providers, and potential and current PrEP users highly ranked community-based HIV testing as a way to facilitate PrEP uptake [36].

#### Adherence Supporters

Healthcare providers were trained to advise all individuals initiating PrEP to select an adherence supporter, defined as “someone in your life who can support you in taking PrEP pills every day and to remind you to get refills.” Providers suggested selecting someone whom the PrEP user was close to, trusted and saw often, and with whom they spent a lot of time. Adherence supporters’ name and contact information were recorded in medical records. This strategy was intended to leverage the power of peer social support in improving adherence [37] and reducing stigma [38], and was based on HIV care guidelines for treatment partners for people with HIV in several countries, including Uganda, that recommend selection of individuals from patients’ social networks to support HIV treatment adherence and care engagement [39]. Selecting an adherence supporter was encouraged but was not a requirement for obtaining PrEP. Similar guidance for HIV treatment partners has shown effects on improved social support and adherence [40]. Research also suggests that HIV treatment partners can motivate adherence and disclosure, decrease internalized stigma, and provide emotional and tangible social support [41–43].

#### Community Workshops

During outreach events, healthcare providers from local facilities led multiple PrEP workshops. The workshops were open to the general community, and for anyone thinking about PrEP, initiating PrEP, and already using PrEP, including adherence supporters. The intervention workshop included education about PrEP, with time for questions, group discussions and skits on PrEP facts and myths, PrEP stigma, and PrEP advocacy skills, in order to empower PrEP users to discuss PrEP with members of their social network. To address misconceptions identified in formative work [19], accurate information about PrEP was conveyed with positively themed messages that emphasized PrEP’s benefits (e.g., “Stay healthy for yourself, your family, and your community–take PrEP!”). We anticipated that community workshops would increase PrEP uptake through increased PrEP awareness and decreased misconceptions and stigma, based on research in sub-Saharan Africa showing that PrEP is acceptable if misconceptions are addressed and effectiveness explained [44–46].

Healthcare providers were trained to be workshop facilitators using a structured manual based on our formative work with healthcare, policy, and community partners [19], as well as prior group HIV prevention interventions in sub-Saharan Africa on parent-adolescent communication and prevention advocacy [47, 48]. Facilitators were trained to use empathetic, non-judgmental, and non-confrontational strategies when answering questions about PrEP, consistent with a Motivational Interviewing-informed counseling style to motivate health behavior change [49]. During the training, specific Motivational Interviewing strategies (open questions, affirmations, reflective listening, offering information, and summaries) were defined and demonstrated, with examples related to the intervention workshop content, after which facilitators role-played with each other.

#### Check-In Calls

Healthcare providers were trained to make check-in calls to PrEP users two weeks post-initiation to answer any questions (e.g., about side effects), to ask if they were still taking PrEP or if they discontinued (and why), and to remind about the next outreach event (for refills). Providers problem-solved with clients to address barriers to continuing PrEP if needed (e.g., discussed side effects as likely temporary). Thereafter, reminder calls were made about one week prior to the next refill. If the PrEP user was not reached, their adherence supporter was called. Although check-in calls are standard of care for PrEP, for this intervention, enhanced check-in calls included an additional call after initiation to address any medical concerns about PrEP side effects or other medical-related issues, and to problem solve about discontinuation. This enhanced check-in strategy was based on prior research in South Africa, in which nurses checked in with PrEP users shortly after PrEP initiation at community-based sites to discuss adherence barriers and ask about any side effects [29].

### Assessment: Preliminary Effectiveness

#### PrEP Cascade

We collected aggregate healthcare facility data on number of people tested for HIV and eligible for PrEP 6-months pre-intervention and during the intervention period (April 2021–Sept 2021 and September 2021–February 2022, respectively); we did not have access to HIV testing and PrEP-eligible clients’ socio-demographic characteristics. We also collected medical records data for de-identified client-level PrEP initiation and refill dates, age, and gender, representing 6-months of refill data for all PrEP users who initiated in the pre-intervention and intervention periods (April 2021–February 2022). These data allowed us to derive a PrEP cascade: number of fisherfolk tested, proportion of those tested who were HIV-negative, proportion of HIV-negative fisherfolk who were eligible for PrEP, proportion of PrEP-eligible fisherfolk who initiated PrEP, and proportion of PrEP initiators who persisted on PrEP. PrEP eligibility was based on the 2016 Ugandan national guidelines for HIV prevention and treatment, which include fisherfolk as a priority population disproportionately burdened by HIV [39], and the 2022 PrEP technical guidelines, which state that individuals “at substantial risk of HIV infection” are eligible for PrEP [7]; many fisherfolk are categorized as “at substantial risk” under these guidelines (e.g., multiple partners, sero-discordant partners, sex workers, high alcohol use, adolescent girls and young women).

Consistent with prior research operationalizations of persistence [28, 50, 51], we defined PrEP persistence in two ways, to capture continuation at 6-months as well as coverage over the 6-month period: proportion of PrEP users who possessed PrEP medications at 180 days post-initiation (based on having a refill date and number of pills that included the 180th day post-initiation); and a PrEP medication possession ratio of at least 80% coverage by PrEP prescriptions over 6 months. (i.e., 144 of 180 days). Because the implementation strategies were intended for the whole intervention community, these data were collected for all PrEP users from the community who initiated PrEP during outreach events or at healthcare facilities.

Primary analyses compared data from before the intervention to during the intervention period in the intervention community. Separate logistic regression models were used to predict the likelihood of PrEP uptake or persistence (i.e., PrEP possession) at 6-months with a time indicator (before vs. during the intervention). PrEP user age and gender were included as covariates in the regression for persistence at 6-months only. Note that we did not have institutional review board approval to access to age and gender for individuals who did not initiate PrEP and thus could not use covariates for the PrEP uptake analysis (or examine uptake by socio-demographic subgroup). Moreover, no PrEP user crossed the 80% medication possession threshold pre-intervention, and logistic regression, which allows for covariates, cannot be used with an undefined odds ratio. Therefore, Fisher’s Exact test was used to evaluate change in the 80% medication possession persistence outcome. Because of the missing pre-intervention (baseline) data for the comparison community, it was not possible to test pre-post differences-in-differences comparisons between the intervention and comparison communities. Thus, in secondary analyses, we explored differences in PrEP initiation and persistence during the intervention using a community indicator (intervention, comparison).

### Qualitative Interviews

Semi-structured interviews were conducted with a convenience sample of 20 PrEP users (10 who continued at 6-months, 10 who discontinued before 6-months), 9 adherence supporters, and 7 key partners (1 nurse, 4 HIV testing counselors, 1 local community peer healthcare worker, 1 community leader representing the fish trade) at the end of the intervention period. Overall and subgroup sample sizes were based on guidance for qualitative interview sample sizes to reach saturation [52]. Participants were referred by the medical implementing partner and interviewed in-person in a private space at the landing site by a research assistant, a Ugandan cis-gender man MPH affiliated with a local academic institution, who audio-recorded the 30-min interview and took notes. Participants were asked what they thought about each strategy in general as well as specifically (e.g., extent to which they thought the workshops increased PrEP knowledge and reduced stigma and misconceptions; relationship with their adherence supporter; helpfulness of check-in calls) and suggestions for improvement; questions were adapted from a related interview guide that previously was used to assess anticipated acceptability of the strategies [19]. Fisherfolk were eligible if they were 18 years-old or older and offered PrEP at an outreach event. Adherence supporters were eligible if they were 18 years-old or older and had agreed to be an adherence supporter for a PrEP user (regardless of whether the PrEP user they were supporting was interviewed). Key partners were eligible if they were involved with PrEP provision (as a medical provider) or if they were a community leader in the intervention community. Participants received an incentive of 20,000 Ugandan Shillings (~ 5USD) for the interview.

Audio files were transcribed and translated into English from Luganda for a directed content analysis [53] that was based on the expanded CFIR [8, 9]. Four transcripts (containing 112 passages) were independently coded by two coders (Ugandan cis-gender man MPH and Ugandan cis-gender man MSW) for interrater reliability using the qualitative data management program Dedoose. Inconsistencies were resolved by the primary principal investigator (American cisgender woman social psychology PhD). After achieving reliability, which was high (Cohen’s Kappa = 0.87), one coder coded the remainder of the transcripts, after which the other coder and primary principal investigator reviewed all codes.

### Cost and Cost-Effectiveness

We determined cost of the implementation strategies as a whole in the intervention community from the provider perspective over 6 months by collecting data in two exclusive categories: (a) Cost of the strategies, including hourly staff salaries (provided by the implementing partner’s human resources department) to cover time spent in a 5-day training, preparing for and conducting workshops, and conducting check-in calls; rentals/space for outreach events (e.g., tent/chairs for workshops); vehicle rental for transport to the landing site; monthly airtime and data costs for check-in calls; and printing and stationery (for training and implementation); and (b) Cost-sharing between the medical implementing partner and project, including person days (e.g., of medical staff already working at outreach events, but who shared effort and resources with the project). Note that time spent implementing the strategies was estimated after the intervention by staff, rather than collected in real-time. We did not include the cost of the entire outreach events (which were already being conducted), such as the cost of healthcare facility staff effort (e.g., medical and laboratory staff for HIV testing), or the cost of PrEP (which was available for free through a government-funded PEPFAR program). We calculated the cost-effectiveness ratio as the total implementation cost/(average treatment effect × sample size), comparing the pre-intervention period to the intervention period in the intervention community. Costs for all three strategies were used to calculate cost and cost effectiveness regarding PrEP persistence, as all three strategies were intended to support persistence; however, costs for check-in calls and adherence supporters were removed from the analysis for initiation, as PrEP users were exposed to these strategies only after initiating PrEP.

### Informed Consent

Semi-structured interview participants provided written informed consent. To decrease administrative burden on the medical implementing partner team, IRB approval was only obtained for healthcare staff to ask a subset of PrEP users (the first 81) to provide written informed consent allowing for access to implementation data from their medical records (i.e., check-in call delivery and adherence supporter selection).

## Results

### PrEP User Characteristics

In the intervention community, about half (49.4%) of the 312 PrEP initiators overall were women, with a higher percentage of women initiating PrEP during the intervention (54.7%, n = 105 of 192) than before the intervention (40.8%, n = 49 of 120), Fisher’s Exact p = 0.02. PrEP initiators on average were 30.4 years-old (SD = 9.0), and people who initiated PrEP were younger on average before the intervention [M (SD) = 29.1 (7.9) years] than during the intervention [M (SD) = 31.2 (9.5) years], t (286) = 2.1, p = 0.03. In the comparison community during the intervention, 38.2% (n = 166 of 435) of PrEP users were women, and the average age was 27.3 (SD = 5.3). In the intervention vs. the comparison community, PrEP users were more likely to be women, 54.7% vs. 38.2%, p < 0.001 (per Fisher’s exact test), and were older on average, 31.2 (9.5) vs. 27.3 (5.3) years, Satterthwaite t = 5.35 (245), p < 0.001.

### Preliminary Effectiveness

Table 2 shows PrEP cascade data, and Table 3 shows statistical tests of differences in PrEP uptake and persistence, all of which indicated large effect sizes for the primary analyses within the intervention community. Within the intervention community over time, the percentage who initiated PrEP (of those eligible) increased from before to during the intervention. Individuals who initiated PrEP during the intervention period were more likely to possess PrEP medications at 6-months post-initiation than were those who initiated PrEP up to 6-months prior to the intervention start-date. PrEP coverage (i.e., medication possession of 80% or higher) was higher during the intervention than prior to the intervention, when no PrEP user was covered at least 80% of the time.

**Table 2:** PrEP cascade in the intervention community (before and during the intervention period) and comparison community (during the intervention period)

	Tested for HIV *N*	Tested HIV-negative %, n	Eligible for PrEP %, n	Initiated PrEP %, n	Persisted on PrEP at 6-months %, n	At least 80% PrEP coverage over 6-months %, n
Intervention community (Pre-Intervention)	182	93.4, 170	83.5, 142	84.5, 120	6.7, 8	0.0, 0
Intervention community (During Intervention)	547	98.7, 540	36.9, 199	96.5, 192	47.9, 92	35.9, 69
Comparison community (During Intervention)	673	93.9, 632	86.1, 544	78.3, 426	34.0, 145	70.4, 300

**Table 3:** PrEP initiation and persistence differences before vs. during the intervention period in the intervention community, and between the intervention vs. comparison communities during the intervention period

PrEP Outcome	Pre vs. during intervention period: intervention community only			During intervention period: intervention community vs. comparison community		
	OR (95% CI)	p value	Cohen’s d	OR (95% CI)	p value	Cohen’s d
Initiation^a^	5.03 (2.08–12.13)	< 0.0001	0.43	7.60 (3.48–16.59)	< 0.0001	0.59
6-month Persistence	12.88 (5.96–27.85)^b^	< 0.0001	1.01	1.78 (1.26–2.52)^c^	0.001	0.32
At least 80% coverage	N/A^d^	< 0.0001	1.28	0.24 (0.16–0.34)^e^	< 0.0001	0.80

^a^Adjusted analyses could not be calculated for initiation (because age and gender were not collected for non-initiators)

^b^Adjusted OR for persistence = 14.81 (6.75–32.69), p < .0001, d = 1.49, controlling for gender [woman OR (CI) = 0.90 (0.53–1.55), p = .72] and age [OR (CI) = 0.96 (0.93—0.99) per year of age, p = .006]

^c^Adjusted OR for persistence = 1.81 (1.26–2.62), p = 0.002, d = 0.33, controlling for gender [woman OR (CI) = 1.29 (0.92–1.81), p = .14] and age [OR (CI) = 0.99 (0.96–1.01) per year of age, p = .23]

^d^p value from Fisher’s Exact test is reported for coverage, rather than an adjusted OR, because 0% of PrEP users had ≥ 80% medication coverage pre-intervention versus 36% of PrEP users during the intervention, resulting in an undefined odds ratio

^e^Adjusted OR for coverage = 0.22 (0.15–0.32), p < .0001, d = 0.84, controlling for gender [woman OR (CI) = 1.33 (0.93–1.90), p = .12] and age [OR (CI) = 1.00 (0.98–1.03) per year of age, p = .78]

In secondary analyses comparing the intervention and comparison communities, a higher percentage of individuals eligible for PrEP initiated PrEP in the intervention than comparison community (a medium effect), and the percentage of PrEP users who showed PrEP possession at 6-months was higher in the intervention than comparison community (a small-to-medium effect). However, a higher percentage of PrEP users in the comparison community showed high PrEP coverage during the intervention (a medium-to-large effect).

### Semi-structured Interview Analysis: Perceived Acceptability

As illustrated by quotes in Table 4, participants in the intervention community had favorable views of the implementation strategies, showing high acceptability. Positive attitudes seemed to span all gender, age, and participant subgroups.

**Table 4:** Intervention acceptability based on qualitative interviews with 20 Fisherfolk (10 who continued PrEP, 10 who discontinued PrEP), 9 adherence supporters, and 7 key partners post-intervention

Implementation strategy (Themes)	Fisherfolk perspective	Key partner perspective
Workshop: effective in increasing PrEP use	Man, continued PrEP: “Those workshops increased [the number of PrEP users) because I was not using but am now among those who are using meaning that in the community I might not have been the only person we might have come out many who started using it—so I think those workshops have helped.”	Woman counselor: “The numbers used to increase every day, every time we give workshops… We find that they come; they first listen to what we're telling them and then they decide positively about starting PrEP. So, those people who really were engaged in workshops, to listen to the message of PrEP, up to now, they are still taking their PrEP.”
Workshop: increased knowledge/reduced misconceptions	Woman, continued PrEP: “Those workshops have helped us a lot because they teach us; you get to know that if you are going to have sex, you’d know how to protect yourself… I have attended like four.”	Man chairperson of fish sellers and buyers: “Workshops in the community are more viable [than one-on-one learning] because many get to ask questions, even those that seem to be off-topic and it ends up benefiting them all.”
	Woman, Adherence Supporter: “They [workshops] are very good because me as a person I learnt what I did not know. I didn’t know that HIV can be prevented, I only knew about the treatment.”	
Workshop: decreased stigma	Man, continued PrEP: “I think [the workshop decreased stigma] because before the workshop, many people thought these drugs were being given to those who were positive but in the workshop we were told that there are those for positive and negative, and for us who attended we are the ones now telling others that this is PrEP and the other one is ARVs”	Man Village Health Team provider (local peer community health worker): “We told them during the workshops that the person taking PrEP is negative and is just protecting themselves. That’s why people realized that there was no need to stigmatize anyone.”
Workshop: promoted PrEP advocacy	Man, continued PrEP: “It worked well because many people came and attended those workshops… Many started taking PrEP but also they went and told their friends to come. Myself I started and encourage my close friends to…attend and they also started”	Woman counselor: “During the workshops… we told people to preach the word…Then when we go back, we find new ones. They say, ‘that is my friend who told me about it and we are coming from such and such an island’… So, you find that the numbers keep on increasing, but it comes from the workshop.”
Adherence supporters (instrumental support/reminders; emotional support/encouragement)	Man, continued PrEP: “[My adherence supporter is] my sister. … So she's aware about everything of mine…She took it upon herself to be reminding me to take PrEP every day. She also told me that I need to quit alcohol or reduce it. … [Adherence supporters] should be trained to talk kindly and convince people that the pill is not an ARV drug, such that they can understand it.”; Female Adherence Supporter: I have made him confident, using myself as an example. I also started on ART…he picks courage from me	Woman counselor: “[Adherence supporters] are really doing good work, because you find that if this person is busy or too busy and the treatment supporter is aware that maybe this person is remaining with one or two tablets, the supporter can call us themselves to inform us about his or her refill. So, by the time the PrEP user is getting to know that the PrEP is getting done, already the other person has called us.”
Check-in calls: showcaring/provide emotional support	Woman, discontinued PrEP: “Whenever you get a phone call from a provider, you feel so loved and you realize [they] care about you and you take responsibility to start encouraging yourself to take the drugs…[Calls] should be made often because at times you might lose hope.”	Woman counselor: “They felt loved, they felt that they are still being thought about and you find that someone is having a good positive attitude about the health workers calling them. So, they were actually happy calling them and they feel at home.”
Suggestions (workshops)	Woman, discontinued PrEP: “I suggest that the workshops are extended to other places so that people are taught that PrEP is for negative people. Otherwise only those who attended the workshops would stop the stigma and yet the majority of people who stigmatize others are out there.”	Man Village Health Team: “We should take these workshops to other fishing communities… We can then empower our people to know what PrEP is; how they understand it, how it works, because many ask about it but they don’t know it.”
	Woman, continued PrEP: “The workshops should be the same, for me who is going to take PrEP and my supporter…what they are reminding you of is not clearly understood by them.”	Woman nurse: “[Adherence supporters need to know] everything about PrEP, because if you choose me…when I know nothing about PrEP, I might sometimes remind you, but … If I know everything about PrEP, why you're taking PrEP, and the benefits of PrEP, for sure I will be able to support you accordingly.”

#### Workshops

Both key partners and fisherfolk said that the workshops were critical in teaching basic facts about PrEP, such as conveying the importance of staying on PrEP to prevent HIV and addressing misconceptions. Fisherfolk expressed that the workshops contributed to reduced anticipated stigma (e.g., fear of discrimination such as gender-based violence) and enacted stigma (e.g., gossip), by promoting open community discussions about and norms for acceptance around PrEP; providers reported similar perceptions. Some fisherfolk attended workshops multiple times and continued to learn, and others said that the workshops led people to advocate for PrEP and counter misinformation in their community. Workshop facilitators expressed that they saw the effectiveness of the workshop firsthand, noting that they saw people who attended workshops initiate and continue PrEP. Some observed that the skits as well as the question-and-answer components were especially helpful in engaging fisherfolk.

Participants across categories suggested scaling-up strategies to bring the workshops to other fisherfolk, including to other landing sites and to islands with less healthcare access. Some fisherfolk who discontinued discussed challenges with getting refills and suggested sponsoring additional outreach events as well as having more healthcare facilities to provide PrEP. Some participants suggested shortening workshop length to 30 min from 1–2 h, so that fisherfolk could stay for the entire session (and not need to leave for work or to get HIV test results) and providing more hands-on practice for facilitators.

#### Check-In Calls

Fisherfolk participants perceived that the check-in calls showed caring by providers, which motivated them to continue PrEP. Providers who conducted the calls confirmed this point, saying that fisherfolk seemed to feel supported by the check-in calls; providers also said that check-in calls could help to address forgetting, a primary adherence barrier. Providers liked that they had the option of reminding adherence supporters if PrEP users could not be reached. Overall, participants were satisfied with the cadence of the check-in calls; some suggested more frequent calls, especially for non-adherent PrEP users.

#### Adherence Supporters

Fisherfolk said that adherence supporters motivated them through emotional support (e.g., encouragement, caring) as well as instrumental support (e.g., reminders, food preparation), and providers echoed these sentiments. Adherence supporters said that they reminded PrEP users in person as well as by phone (although they had varying success reaching them by phone). Providers said that some fisherfolk expressed that non-disclosure of PrEP use due to stigma could be a barrier to choosing adherence supporters. Participants overall expressed interest in having workshops specifically for adherence supporters, to ensure they were prepared and could practice supportive communication skills.

### Implementation Fidelity

During the intervention period, 31 workshops were conducted with 327 fisherfolk (166 men, 161 women) over 6 outreach events. Observers rated workshop fidelity in real-time on coverage of key elements of content (e.g., role plays) and process (e.g., introductions) as *not at all* (1), *somewhat* (2), or *completely* (3). Average coverage was rated as close to “completely” [M (SD) = 2.76 (0.2)]. Of the 81 PrEP users for whom implementation fidelity data were entered into medical records, most (n = 71, 88%) received check-in calls, 47 (58%) chose an adherence supporter, and 6 (7%) declined to choose an adherence supporter; providers did not record adherence supporter data for 28 (35%) PrEP users.

### Cost and Cost-Effectiveness

The total cost incurred for all three implementation strategies combined over 6 months was $6,529. The cost dropped to $5,160 over 6 months for the community workshops only (to support PrEP initiation), excluding adherence supporter and check-in call costs (which were incurred after PrEP initiation). Based on an intervention impact for PrEP initiation of 0.12, with an additional 23 individuals estimated to have a positive initiation outcome during (vs. before) the intervention, the cost of the strategies for each additional person initiating PrEP would be $224. Based on an intervention impact for PrEP persistence (i.e., medication possession at 6-months) of 0.41, with an additional 38 PrEP users estimated to have a positive persistence outcome of medication possession at 6-months during the intervention, the cost for each additional person persisting on PrEP for 6 months would be $173. Based on an intervention impact of 0.36 for at least 80% PrEP coverage over 6-months, with an additional 69 PrEP users estimated to have a positive persistence outcome of 80% coverage during the intervention, the cost for each additional person persisting on PrEP for 6 months would be $95.

## Discussion

In this pilot test of PrEP community-based implementation, we found preliminary effects for increased PrEP uptake, persistence, and coverage in one fisherfolk mainland site, using a pre-post quasi-experimental design. Prior research has similarly found high PrEP uptake in communities and subgroups with high HIV incidence and prevalence, although discontinuation rates have been high [21, 28, 54]. Our research extends prior work by advancing implementation strategies that may not only lead to increased PrEP uptake, but improved PrEP continuation as well. Nevertheless, persistence rates remained low, suggesting that additional support is needed to bolster persistence among highly mobile populations (although the extent to which PrEP users discontinued because they were truly no longer at risk is unknown).

Notable differences between the originally proposed intervention and comparison community, and the absence of pre-intervention data in the comparison community, precluded our ability to fully test for differences between communities, resulting in imperfect, mismatched comparisons; thus, caution should be used when interpreting these secondary findings. Because the comparison community had greater healthcare access, community members could obtain PrEP refills more easily outside of healthcare outreach events. Accordingly, we found that the comparison community fared better than the intervention community on PrEP persistence when operationalized in terms of a medication possession ratio of 80% coverage or greater. However, the intervention community had better persistence when defined as medication possession at the 6-month mark. Thus, our implementation strategies may have helped with long-term PrEP persistence in this highly mobile population with low healthcare access but were unable to overcome barriers to obtaining refills in the shorter-term.

Our intervention was based on current practice and developed in partnership with the Uganda Ministry of Health, which may increase its feasibility for future implementation. The adherence supporter strategy was adapted from treatment partner interventions for people with HIV, a policy codified in HIV care guidelines of multiple countries, including Uganda [39]. Our workshops extended community-based healthcare outreach events that are a staple of HIV prevention and treatment services in low-and-middle-income countries [32, 55]. The workshops, which adapted some content from prior effective HIV prevention research interventions [47, 48], enhanced standard community outreach strategies by tailoring discussions about PrEP to address common community myths, and providing education about PrEP, with time for facilitators to answer questions in a non-judgmental style, as well as skits to foster skills to advocate for PrEP. The check-in calls, a strategy also adapted from prior research [29], similarly enhanced existing standard of care, by providing scripted guidance to healthcare providers for initial inquiries with PrEP users shortly after initiation, in addition to refill reminders. It is important to note that only oral PrEP was available at the time of the study, and long-acting injectable PrEP would need different implementation strategies [56].

Although our available data suggested high fidelity to the workshop and check-in call protocols, there was only moderate feasibility for adherence supporter implementation: When asked, only a small percentage of PrEP users declined to select an adherence supporter, but about a third of PrEP users were missing data for adherence supporters, suggesting that they were not asked to select one. Our fidelity assessment was limited, as fidelity for check-in calls and adherence supporters were only assessed at the beginning of the intervention period.

Qualitative interview data helped to uncover mechanisms that may have led to higher PrEP initiation and persistence in the intervention community over time. Consistent with PrEP determinants hypothesized under expanded CFIR conceptualizations [8, 9], such behavior change mechanisms included decreased stigma and increased social support, as well as increased positive community norms for PrEP. However, we did not conduct dyadic interviews to understand adherence supporters’ relationship dynamics that could have led to behavior change. In addition, those who discontinued had similarly positive attitudes about the strategies as did those who continued, and thus, the strategies may not have adequately addressed common barriers to HIV services (including PrEP) indicated by prior fisherfolk research, including competing work responsibilities (which can impede refill collection), side effects, and partner issues (e.g., unsupportive partner) [15, 16, 18, 19]. Moreover, we did not quantitatively assess these potential mechanisms of behavior change, and participants’ perceptions could have been clouded by social desirability biases.

Our study has implications for community-based PrEP delivery in other contexts with high HIV prevalence and moderate rates of HIV viral suppression. When PrEP is de-stigmatized and demystified, with open factual discussions to counteract myths and concerns, people may recognize its value. They also may feel more comfortable taking PrEP over the long-term if members of their communities, including their sexual partners, are similarly exposed to PrEP education and thus understand the value of PrEP as a strong prevention tool.

The per-person cost of the strategies for PrEP uptake was higher than the per-person cost for PrEP persistence. Differences were due in large part to already high initiation rates for our healthcare outreach events, in the absence of the other implementation strategies. Although the strategies may be worth the cost in improving persistence, the value of the implementation strategies may be incremental for increasing initiation, which potentially may be increased through less intensive means for sensitizing communities about PrEP through outreach events.

In sum, our study showed encouraging findings for augmenting PREP provision with implementation strategies based on behavior change principles. Future effectiveness-implementation research is needed to test these strategies in a cluster randomized trial that includes settings with less access to care (e.g., island communities). For optimal outcomes in generalized HIV epidemics, it is critical to develop implementation strategies for PrEP that encompass and engage whole communities.

## Data Availability

All authors ensure that the data support the published claims and comply with field standards.
